# Supplementary material for: Morphological evolution of bird wings follows a mechanical sensitivity gradient determined by the aerodynamics of flapping flight
Source: Nat Commun. 2023 Nov 18;14:7494. doi: 10.1038/s41467-023-43108-2 (PMC10657351; doi:10.1038/s41467-023-43108-2)
Supplement: Supplementary file 1 — Supplementary Information [file 41467_2023_43108_MOESM1_ESM.pdf]

Supplementary Materials for:

Morphological evolution of bird wings follows a mechanical sensitivity gradient determined by the aerodynamics of flapping flight

Supplementary Note 1

Mechanical sensitivity models for flight

Here we elaborate on the underlying mechanical sensitivity models for the wings of flying animals described in the main text. Mechanical sensitivity relates the rate of change in some aspect of morphology to the rate of change in a relevant biomechanical output. Here we select aerodynamic lift as the most relevant biomechanical parameter for flight. All flying animals must generate lift sufficient to support their body weight, whereas other potentially relevant aerodynamic parameters such as drag and the mechanical power required for flight are not similarly constrained, making them less than ideal candidates for these models. However, variation in overall body size means that the total amount of lift differs among birds, so a general model of mechanical sensitivity must be normalized as a percent change in lift for a percent change in morphological properties.

Flapping

We model mechanical sensitivity in flapping flight following the strips (i.e. blade-element) analysis popularized by Weiss-Fogh [1]. In brief, this analysis divides the wing into a set of infinitesimally thin chord-wise strips and integrates the lift produced by each strip along the root to tip length of the wing to compute the total. The lift produced by an airfoil is calculated as:

$$L = \frac{1}{2}\rho\, S\, u^2\, C_l \qquad \text{Eqn. S1}$$

where L is lift, S is airfoil area, u is flow velocity past the airfoil, and C_l is the coefficient of lift, a non-dimensional quantity. We use a subscript i to denote the lift from the ith section, and express area S as the product of the section width dr and section chord length c_i.

$$L_i = \frac{1}{2}\, \rho\, dr\, c_i\, {u_i}^2\, C_{l,i} \qquad \text{Eqn. S2}$$

Equation S2 provides the lift for each strip in terms of the flow velocity u_i past the strip. For flapping flight, this flow velocity also varies from base to tip due to the motion of the wing. For hovering flight, velocity is zero at the base, reaches a maximum at the tip, and is given by:

$$u_{(i,t)} = r_i \dot{\theta}_t \qquad \text{Eqn. S3}$$

where $\dot{\theta}_t$ is the instantaneous angular velocity of flapping.

Substitution, of Eqn. S3 into Eqn. S2 and averaging over a full flapping cycle produces:

$$L_i = \frac{1}{2}\, \rho\, dr\, c_i\, {r_i}^2\, \overline{\dot{\theta}^{\,2}}\, C_{l,i} \qquad \text{Eqn. S4}$$

where $\overline{\dot{\theta}^{\,2}}$ is the average squared angular velocity. This result could then be integrated over the wing radius to produce a total estimate of lift:

$$L = \frac{1}{2}\, \rho\, \overline{\dot{\theta}^{\,2}}\, R \int_0^1 c(\hat{r})\; \hat{r}^2\, C_l(\hat{r})\, d\hat{r} \qquad \text{Eqn. S5}$$

where $\hat{r}$ is the non-dimensional radius, i.e. the actual radius r_i of the strip divided by the length of the wing, R. Equations S4 and S5 show that for a wing of length R, the percent change in lift at a particular spanwise section i or non-dimensional spanwise location $\hat{r}$ produced by a change in chord or coefficient of lift depends on $\hat{r}^2$. For example, increasing C_l by 20% at $\hat{r} = 0.8$ versus $\hat{r} = 0.4$ produces ${0.8^2}/{0.4^2} = 4$ times the proportional increase in lift for that section, which therefore has 4 times greater mechanical sensitivity for changes in chord c or coefficient of lift C_l. This produces the following curve:

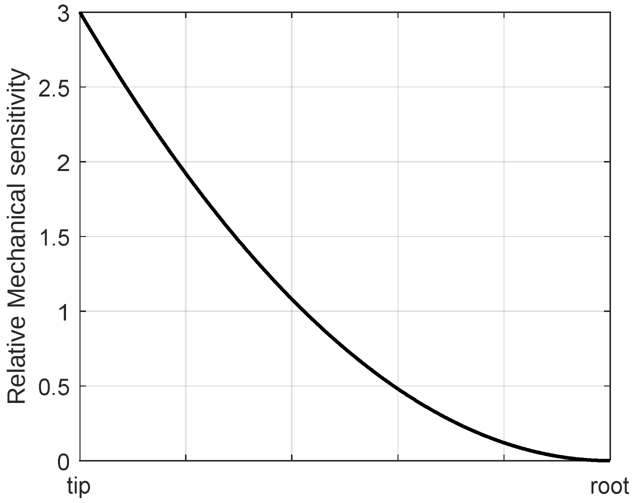

Figure S1: mechanical sensitivity in flapping. This curve shows the predicted mechanical sensitivity of wing sections from root to tip. Panel A is strictly in terms of $\hat{r}$, while panel B has the result multiplied by three so that the average value of the curve is 1.0 to simplify comparison with gliding wings.

Figure S1 directly predicts the wing section mechanical sensitivity for quantities such as chord that have a direct, linear relationship with lift in Eqns. S4 and S5. However, of the four quantities we measured: camber, chord, cross-sectional area, and cross-sectional thickness, only chord meets this criterion. The other three parameters: camber, cross-sectional area, and cross-sectional thickness are all expected to affect the coefficient of lift C_l, but may not have a simple linear relationship with it. However, assuming that the relationship, whatever it is, is similar among all wing sections, the underlying mechanical sensitivity curve will be as shown in Fig. S1.

Flapping wing aerodynamics are substantially more complex than can be described by the simplified model in Eqns. S1-5, and depend on the formation of and interactions between the tip vortex, leading edge vortex, and trailing edge vortex [2]. Despite this, the underlying base to tip force gradient remains a prominent feature of complex numerical simulations of flapping flight that natively incorporate vortex dynamics [2].

Inertia in flapping

When birds flap their wings, they must use mechanical power to overcome the inertia of the wing, the cost of which depends on the moment of inertia of the wing as well as flapping frequency and amplitude [3]. Thus, wing moment of inertia might also produce a mechanical sensitivity gradient in the wings of flapping flyers. The moment of inertia for a wing flapping about its base is given by:

$$M = R \int_0^1 m(\hat{r})\ \hat{r}^2\ d\hat{r} \qquad \text{Eqn. S6}$$

Where $m(\hat{r})$ is the mass of the wing at non-dimensional spanwise location $\hat{r}$. Note that this equation produces the same general mechanical sensitivity curve that is created by the aerodynamics of flapping (Fig. S1), but depends on wing mass instead of wing chord or coefficient of lift. Thus, considering inertia as well as lift does not alter the overall mechanical sensitivity of flapping flight, and these effects may work synergistically to form a gradient of mechanical sensitivity along the wing.

References

1. Weis-Fogh, T. Energetics of Hovering Flight in Hummingbirds and in *Drosophila*. *Journal of Experimental Biology* **56**, 79–104 (1972).
2. Shyy, W. *et al.* Recent progress in flapping wing aerodynamics and aeroelasticity. *Progress in Aerospace Sciences* **46**, 284–327 (2010).
3. van den Berg, C. & Rayner, J. M. V. The moment of inertia of bird wings and the inertial power requirement for flapping flight. *J Exp Biol* **198**, 1655–1664 (1995).

Supplementary Note 2

Supplementary Figures

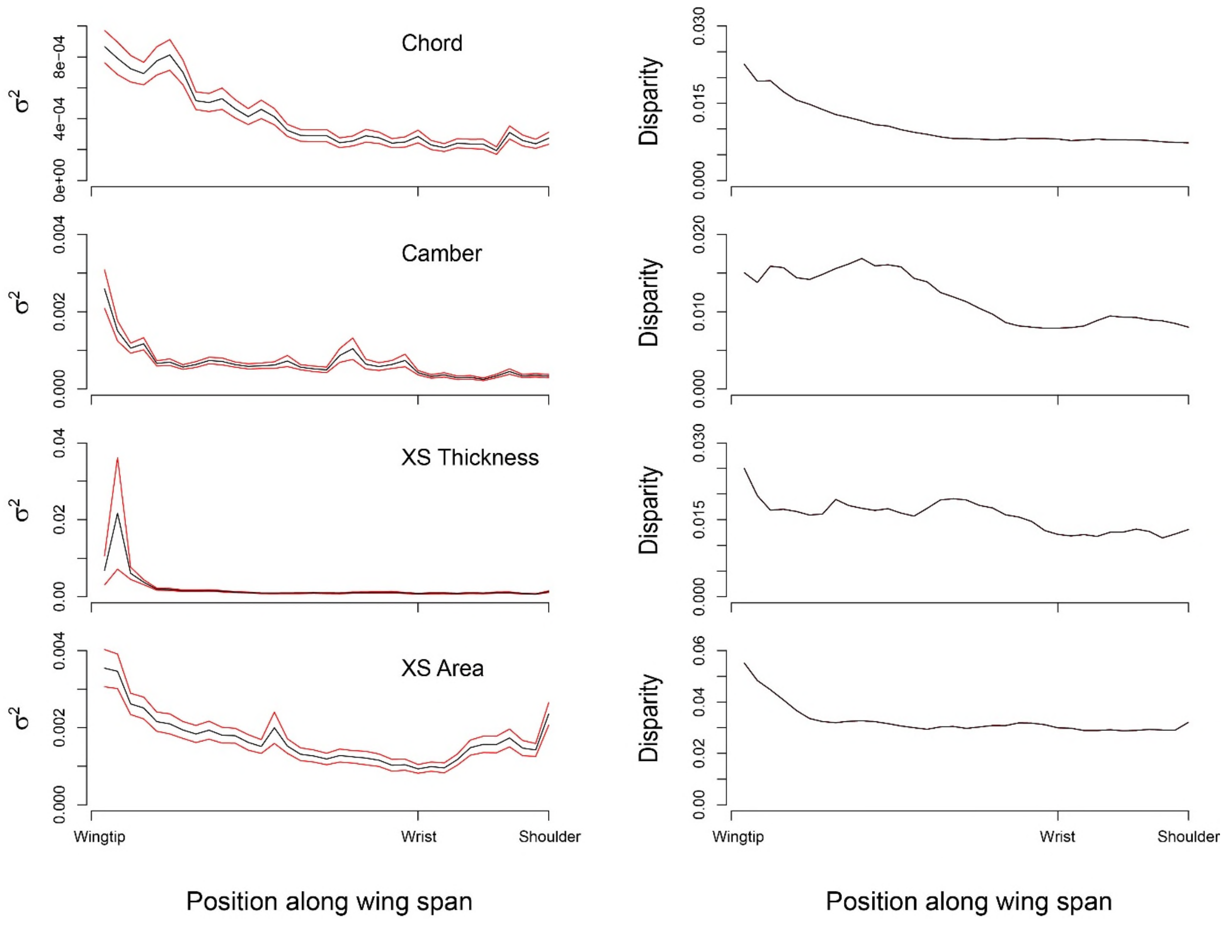

Figure S2. We iterated our analysis across 1000 posterior draws of the bird tree with varying topologies to assess the effect of tree topology on our estimates of σ^2 (left panes) and disparity (right panes). The black lines depict medians of the estimated values along the wing for 1096 wings from 178 species, with the red lines showing the median absolute deviation (MAD) bounds. Evolutionary rate is sensitive to changes in tree topology, however the general pattern of greater σ^2 at the wingtip is maintained across the tested topologies. Disparity, unlike σ^2, was insensitive to tree topology.

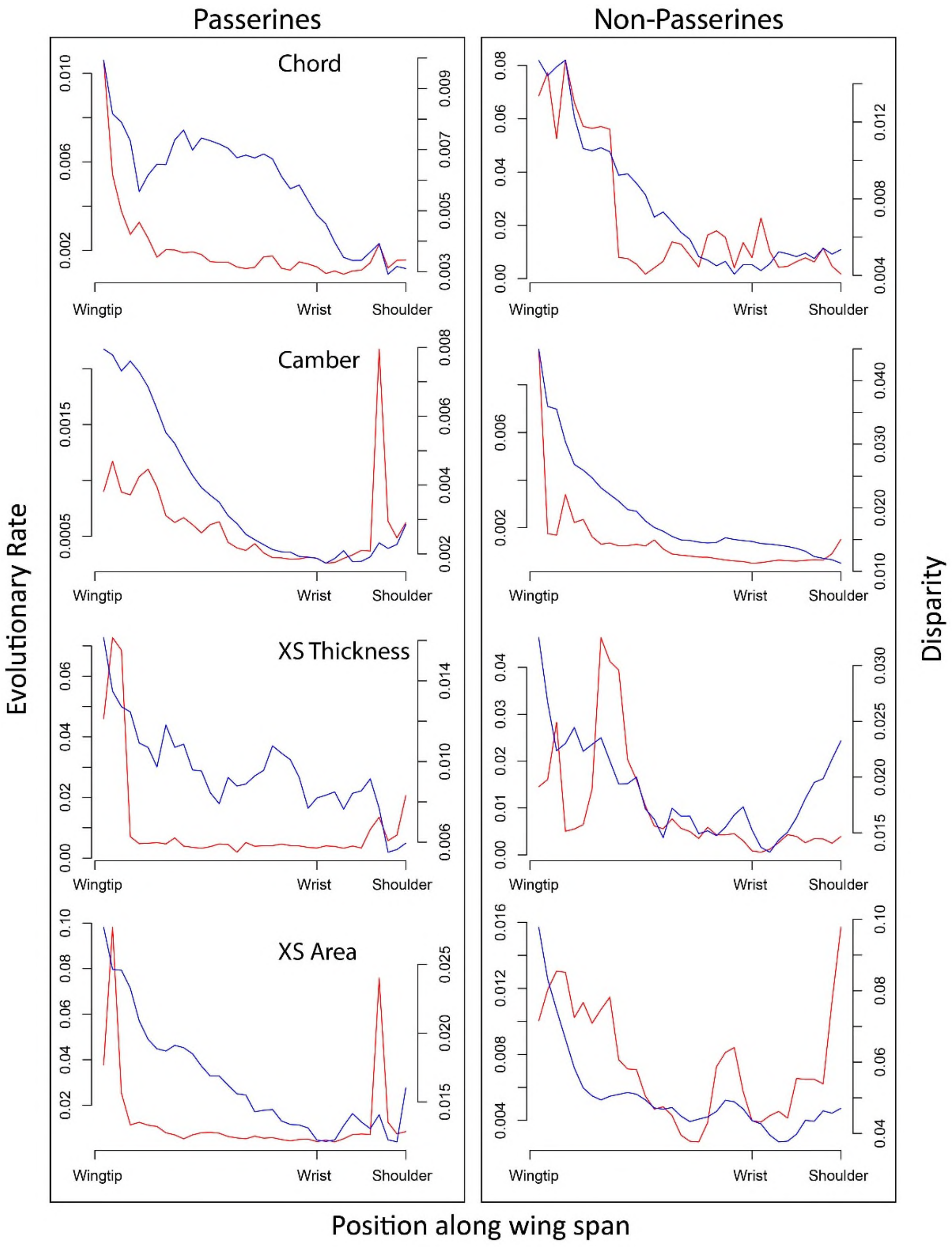

Figure S3. The gradient patterns of both σ^2 (red lines) and disparity (blue lines) were consistent across different partitions of the data. Both passerine (113 species; left panes) and non-passerine birds (65 species; right panes) showed similar patterns of σ^2 and disparity.

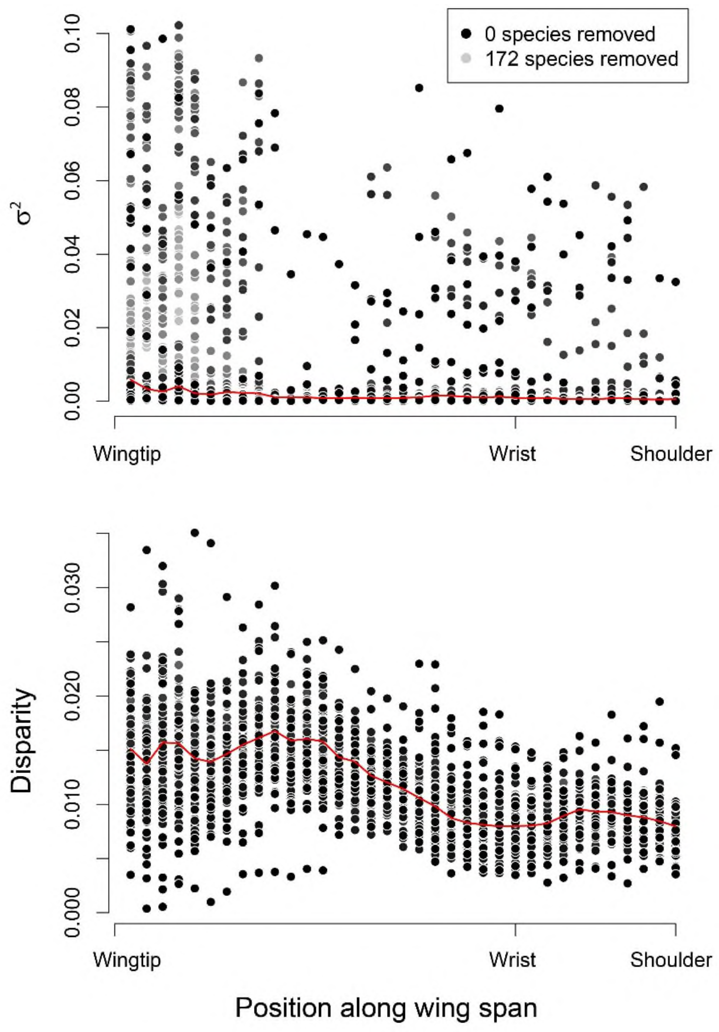

Figure S4. We conducted a rarefaction analysis to test the robustness of the of our main finding to our phylogenetic sample. We randomly removed from 1 to 172 (of 178) species (with replacement) and recalculated evolutionary rate σ^2 and morphological disparity. Points show measured σ^2 (top pane) and disparity (bottom pane) at each wing slice for each rarefied data set. Red lines show median values among the rarefied data sets.

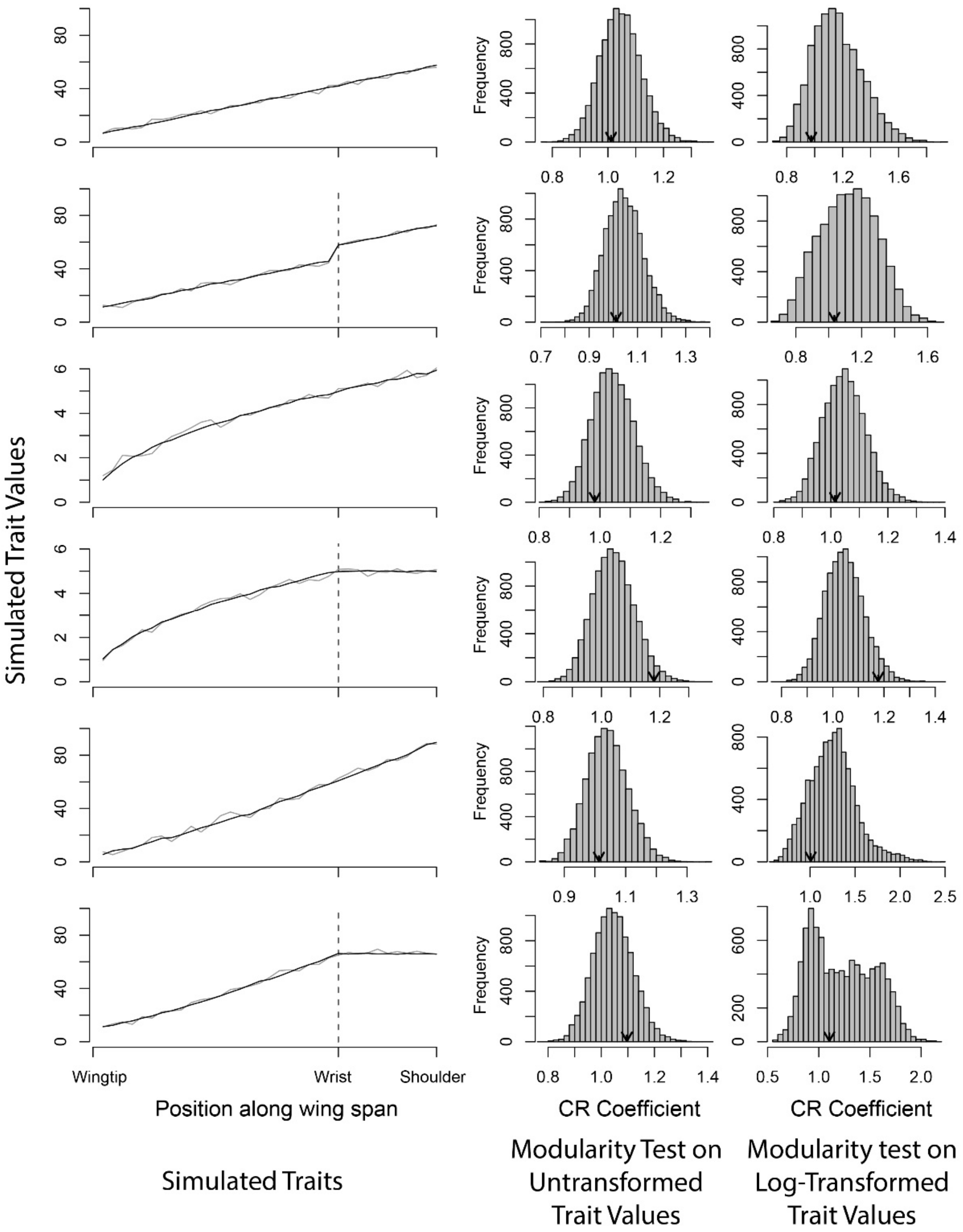

Figure S5. We simulated wing trait data (with normal distributions) for 40 theoretical taxa at 35 "slices" along the length of the wing, mirroring our empirical data set, with six different underlying trends. Three trends were continuous across the wrist, and three had discontinuities at the wrist (denoted by vertical dashed lines). We then conducted the CR analysis on both the untransformed simulated data and on log-transformations of the data (as in our empirical analysis). None of the CR values were significantly different from 1.0 in our simulations.
