# Supplementary material for: Morphological evolution of bird wings follows a mechanical sensitivity gradient determined by the aerodynamics of flapping flight
Source: Nat Commun. 2023 Nov 18;14:7494. doi: 10.1038/s41467-023-43108-2 (PMC10657351; doi:10.1038/s41467-023-43108-2)
Supplement: Supplementary file 3 — Reporting Summary [file 41467_2023_43108_MOESM3_ESM.pdf]

nature portfolio

Corresponding author(s): Jonathan A. Rader

Last updated by author(s): Oct 9, 2023

Reporting Summary

Nature Portfolio wishes to improve the reproducibility of the work that we publish. This form provides structure for consistency and transparency in reporting. For further information on Nature Portfolio policies, see our Editorial Policies and the Editorial Policy Checklist.

Statistics

For all statistical analyses, confirm that the following items are present in the figure legend, table legend, main text, or Methods section.

n/a	Confirmed	
☐	☒	The exact sample size (n) for each experimental group/condition, given as a discrete number and unit of measurement
☐	☒	A statement on whether measurements were taken from distinct samples or whether the same sample was measured repeatedly
☐	☒	The statistical test(s) used AND whether they are one- or two-sided<br>*Only common tests should be described solely by name; describe more complex techniques in the Methods section.*
☐	☒	A description of all covariates tested
☐	☒	A description of any assumptions or corrections, such as tests of normality and adjustment for multiple comparisons
☐	☒	A full description of the statistical parameters including central tendency (e.g. means) or other basic estimates (e.g. regression coefficient) AND variation (e.g. standard deviation) or associated estimates of uncertainty (e.g. confidence intervals)
☐	☒	For null hypothesis testing, the test statistic (e.g. F, t, r) with confidence intervals, effect sizes, degrees of freedom and P value noted<br>*Give P values as exact values whenever suitable.*
☒	☐	For Bayesian analysis, information on the choice of priors and Markov chain Monte Carlo settings
☒	☐	For hierarchical and complex designs, identification of the appropriate level for tests and full reporting of outcomes
☒	☐	Estimates of effect sizes (e.g. Cohen's d, Pearson's r), indicating how they were calculated

Our web collection on statistics for biologists contains articles on many of the points above.

Software and code

Policy information about availability of computer code

Data collection	We used a NextEngine 3D scanner to digitize shape data from 1096 bird wings from 178 species of birds. We developed a custom Matlab program to quantify shape variable from the scanned wings.<br><br>All shape trait data and the phylogenetic tree generated in this study have been deposited, without restrictions, in figshare under the accession code dx.doi.org/10.6084/m9.figshare.16899580
Data analysis	We used R version 4.1.3 (2022-03-10) for statistical analyses of the data.<br><br>All analysis scripts are available at dx.doi.org/10.6084/m9.figshare.16899580

For manuscripts utilizing custom algorithms or software that are central to the research but not yet described in published literature, software must be made available to editors and reviewers. We strongly encourage code deposition in a community repository (e.g. GitHub). See the Nature Portfolio guidelines for submitting code & software for further information.

Data

Policy information about availability of data

All manuscripts must include a data availability statement. This statement should provide the following information, where applicable:

- Accession codes, unique identifiers, or web links for publicly available datasets
- A description of any restrictions on data availability
- For clinical datasets or third party data, please ensure that the statement adheres to our policy

All shape trait data and the phylogenetic tree generated in this study have been deposited, without restrictions, in figshare under the accession code dx.doi.org/10.6084/m9.figshare.16899580

Research involving human participants, their data, or biological material

Policy information about studies with human participants or human data. See also policy information about sex, gender (identity/presentation), and sexual orientation and race, ethnicity and racism.

Reporting on sex and gender	n/a
Reporting on race, ethnicity, or other socially relevant groupings	n/a
Population characteristics	n/a
Recruitment	n/a
Ethics oversight	n/a

Note that full information on the approval of the study protocol must also be provided in the manuscript.

Field-specific reporting

Please select the one below that is the best fit for your research. If you are not sure, read the appropriate sections before making your selection.

☐ Life sciences ☐ Behavioural & social sciences ☒ Ecological, evolutionary & environmental sciences

For a reference copy of the document with all sections, see nature.com/documents/nr-reporting-summary-flat.pdf

Ecological, evolutionary & environmental sciences study design

All studies must disclose on these points even when the disclosure is negative.

Study description	We used first-principles physics to make predictions about the evolutionary divergence of wing shape in birds. We quantified three-dimensional wing shape among a broad sample of bird species and used phylogenetic analyses to test our predictions.
Research sample	We sampled 1096 preserved wings from 178 species of birds. The wings were prepared by and are stored at the North Carolina Museum of Natural Sciences in Raleigh, NC. Our sample was chosen to maximize taxonomic breadth to allow our results to broadly reflect avian evolutionary history. Our sample is drawn largely from collections of North American birds.
Sampling strategy	We were interested in broad, Ayes-wide patterns of evolution. To facilitate this level of analysis, we assembled the a broad taxonomic sample. Species-level sample sizes were subject to availability within the museum collections.
Data collection	Data were collected using a NextEngine 3D laser scanner, operated by the corresponding author and a number of lab technicians.
Timing and spatial scale	Museum wing specimens were scanned between 2017 and 2020. The project commenced in 2017 and was ended when the COVID-19 pandemic imposed restrictions on access to the research collections at the North Carolina Museum of Science. The collection - and our sample- mainly contains samples from the southeastern U.S., but also has samples from across the rest of North America as well as Europe and South America.
Data exclusions	The species "Phalaropustricolor" was scanned, but excluded from analysis as it was not present in the phylogenetic tree that we used for comparative analyses.
Reproducibility	Measures were taken to ensure reproducibility at various stages in the analysis pipeline, including testing to ensure that wings scanned at different resolutions produced similar results, by post-processing of the wing shape data into fewer or greater slices, and verifying that overall conclusions were robust to the removal of individual species from the analysis. These measures were taken initially during development of the scanning methods. We also conducted a rarefaction analysis to test the sensitivity of our results to the inclusion or exclusion of various taxa - we found at all stages that our particular choices of methodology or sampling had little effect on finding our main results, which we therefore suggest are robust such factors.

Randomization	When possible, i.e. when sufficient specimens were available, specimens were randomly sampled from the museum collection for scanning. However, this was an uncommon occurrence and for most species, all available specimens were included.
Blinding	The study was not blinded, and the researchers collecting the scans were aware of the species of the wings they were scanning, though not necessarily the phylogenetic placement of that species.

Did the study involve field work? ☐ Yes ☒ No

Reporting for specific materials, systems and methods

We require information from authors about some types of materials, experimental systems and methods used in many studies. Here, indicate whether each material, system or method listed is relevant to your study. If you are not sure if a list item applies to your research, read the appropriate section before selecting a response.

Materials & experimental systems

n/a	Involved in the study	
☒	☐	Antibodies
☒	☐	Eukaryotic cell lines
☒	☐	Palaeontology and archaeology
☒	☐	Animals and other organisms
☒	☐	Clinical data
☒	☐	Dual use research of concern
☒	☐	Plants

Methods

n/a	Involved in the study	
☒	☐	ChIP-seq
☒	☐	Flow cytometry
☒	☐	MRI-based neuroimaging

Plants

Seed stocks	n/a
Novel plant genotypes	n/a
Authentication	n/a
